# Antitumor activity of the ERK inhibitor SCH722984 against BRAF mutant, NRAS mutant and wild-type melanoma

**DOI:** 10.1186/1476-4598-13-194

**Published:** 2014-08-20

**Authors:** Deborah JL Wong, Lidia Robert, Mohammad S Atefi, Amanda Lassen, Geetha Avarappatt, Michael Cerniglia, Earl Avramis, Jennifer Tsoi, David Foulad, Thomas G Graeber, Begonya Comin-Anduix, Ahmed Samatar, Roger S Lo, Antoni Ribas

**Affiliations:** Department of Medicine, Division of Hematology-Oncology, University of California Los Angeles (UCLA), 11-934 Factor Building, Los Angeles, CA USA; Department of Molecular and Medical Pharmacology, UCLA, Los Angeles, CA USA; Department of Surgery, Division of Surgical-Oncology, UCLA, Los Angeles, CA USA; Jonsson Comprehensive Cancer Center at UCLA, 10833 Le Conte Avenue, Los Angeles, CA 90095-1782 USA; Discovery Oncology Merck Research Laboratories, Merck Research Laboratories, Boston, Massachusetts USA; Department of Medicine, Division of Dermatology, UCLA, Los Angeles, California USA; University of Applied Sciences, Vienna, Austria

**Keywords:** ERK inhibitor, BRAF inhibitor, Melanoma, Targeted therapy, Acquired resistance

## Abstract

**Background:**

In melanoma, dysregulation of the MAPK pathway, usually via *BRAF*^*V600*^ or *NRAS*^*Q61*^ somatic mutations, leads to constitutive ERK signaling. While BRAF inhibitors are initially effective for *BRAF*-mutant melanoma, no FDA-approved targeted therapies exist for BRAF-inhibitor-resistant *BRAF*^*V600*^, *NRAS* mutant, or wild-type melanoma.

**Methods:**

The 50% inhibitory concentration (IC50) of SCH772984, a novel inhibitor of ERK1/2, was determined in a panel of 50 melanoma cell lines. Effects on MAPK and AKT signaling by western blotting and cell cycle by flow cytometry were determined.

**Results:**

Sensitivity fell into three groups: sensitive, 50% inhibitory concentration (IC_50_) < 1 μM; intermediately sensitive, IC_50_ 1-2 μM; and resistant, >2 μM. Fifteen of 21 (71%) *BRAF* mutants, including 4 with innate vemurafenib resistance, were sensitive to SCH772984. All three (100%) *BRAF/NRAS* double mutants, 11 of 14 (78%) *NRAS* mutants and 5 of 7 (71%) wild-type melanomas were sensitive. Among *BRAF*^*V600*^ mutants with *in vitro* acquired resistance to vemurafenib, those with MAPK pathway reactivation as the mechanism of resistance were sensitive to SCH772984. SCH772984 caused G1 arrest and induced apoptosis.

**Conclusions:**

Combining vemurafenib and SCH722984 in BRAF mutant melanoma was synergistic in a majority of cell lines and significantly delayed the onset of acquired resistance in long term *in vitro* assays. Therefore, SCH772984 may be clinically applicable as a treatment for non-*BRAF* mutant melanoma or in *BRAF*-mutant melanoma with innate or acquired resistance, alone or in combination with BRAF inhibitors.

**Electronic supplementary material:**

The online version of this article (doi:10.1186/1476-4598-13-194) contains supplementary material, which is available to authorized users.

## Background

Currently, targeted therapy for metastatic melanoma hinges on determining *BRAF* mutational status. Approximately 50% of all melanomas contain an activating *BRAF*^*V600*^, a serine-threonine kinase which functions as an oncogenic driver. This constitutively activates the MAPK pathway via sequential phosphorylation of MEK and then ERK. Vemurafenib and dabrafenib, two type I RAF inhibitors, selectively inhibit *BRAF*^*V600*^, leading to improvements in both progression free survival and overall survival for patients with this disease [1–3]. However, BRAF inhibition is not effective in the remaining 50% of *BRAF* wild-type melanoma, including *NRAS*^*Q61*^ melanoma. Indeed, treatment of non-BRAF mutant cells with dabrafenib or vemurafenib would result in paradoxical activation of the MAPK pathway, mediated by CRAF [4, 5].

For *BRAF*^*V600*^ mutant melanoma, initial response rate to BRAF inhibitors (BRAFi) is beyond 50%, though median duration of response is only 6-7 months. Resistance to BRAFi has been reported to occur via MAPK-dependent and -independent mechanisms. Reported MAPK-dependent mechanisms include secondary mutations in *NRAS* [6] or *MEK* [7, 8], upregulation of the COT pathway [9], mutant *BRAF* gene amplification [10] or development of BRAF^V600E^ splice variants [11]. MAPK-independent mechanisms of acquired resistance also occur, through the upregulation of receptor tyrosine kinases (RTKs), such as the platelet-derived growth factor beta (PDGFRβ) [6], or the insulin growth factor receptor 1 (IGF1R), or deletions of *PTEN* [12]. These all lead primarily to enhanced PI3K/AKT/mTOR signaling rather than reactivation of the MAPK pathway. These mechanisms of acquired resistance are generally mutually exclusive and predict for susceptibility to inhibition with other targeted therapies *in vitro*; cell lines with secondary *NRAS*^*Q61*^ mutations remain sensitive to a MEK inhibitor [13] (MEKi), while cell lines displaying RTK upregulation are cross-resistant to a MEKi but sensitive to a PI3K, AKT or mTOR inhibitor in combination with vemurafenib [14, 15].

Combining BRAFi and MEKi delays the development of resistance *in vitro* compared to treatment with BRAFi or MEKi alone [16, 17]. Likewise, a phase I/II clinical trial of dabrafenib and trametinib in *BRAF*^*V600*^ mutant metastatic melanoma resulted in progression-free survival of 9.4 months compared to 5.8 months in patients treated with dabrafenib alone. Response rates for the combination and dabrafenib alone treatments were 76% and 54%, respectively [2]. However, resistance develops both *in vitro* and *in vivo* to this combination, thus additional treatments for melanoma are needed.

MEKi may have clinical activity in *NRAS* mutant melanoma. *In vitro*, inhibition occurs at nanomolar concentrations [13]. Resistance to MEKi is MAPK dependent [18–20]. *In vivo*, MEK inhibitors are effective in *NRAS*-mutated melanoma, though therapeutic activity is modest compared to BRAFi in *BRAF* mutant melanoma [21, 22].

Inhibition of ERK1 and ERK2 (ERK1/2) is a promising strategy to address both innate and acquired resistance to BRAFi and MEKi, regardless of the upstream mechanism(s) of MAPK reactivation. ERK1/2, the main downstream effectors of the MAPK pathway, activate proteins such as RSK and transcription factors needed to regulate cellular growth and survival [23, 24] such as cyclin D1, which promotes progression through the G1 phase of the cell cycle [25]. There is extensive crosstalk between MAPK and PI3K/AKT pathways [26]. While some data indicate that activation of the MAPK pathway may decrease AKT signaling [27], cross-activation of the PI3K/AKT/mTOR pathway has been shown to be mediated directly by activation of ERK or via activation of RSK, leading to activation of mTORC1 [28, 29]. Cross-inhibition versus cross-activation may vary based on cellular context or be timing dependent. Therefore, inhibiting ERK may result in inhibition of the oncogenic MAPK signaling in most melanomas, with added effects of partially inhibiting proliferative signals through the PI3K/AKT/mTOR pathway.

SCH772984 is a potent, ATP competitive and non-competitive inhibitor of ERK 1/2, with additional allosteric properties that inhibit ERK activation/phosphorylation by MEK [30]. It has been shown to be effective at nanomolar concentrations in multiple tumor cell lines including breast, colon, and melanoma [30]. SCH772984 specificity for ERK1/2 kinases occurs at concentrations up to 1 μM and it inhibits phosphorylation of downstream ERK targets such as RSK. Given its specificity for ERK and the potential for ERK inhibition to inhibit both MAPK and PI3K/AKT pathways, we evaluated the susceptibility of wild-type, mutant BRAF- or NRAS-melanoma, and BRAF-mutant melanoma with acquired BRAFi resistance. We also tested the effect of combined BRAF and ERK inhibition on BRAF-mutant melanoma in short-term and long-term cultures to determine if combination therapy would result in improved inhibition or delay the development of resistance *in vitro*.

## Results

### BRAF-mutant melanoma cell lines are sensitive to ERK inhibition

**Figure 1:**
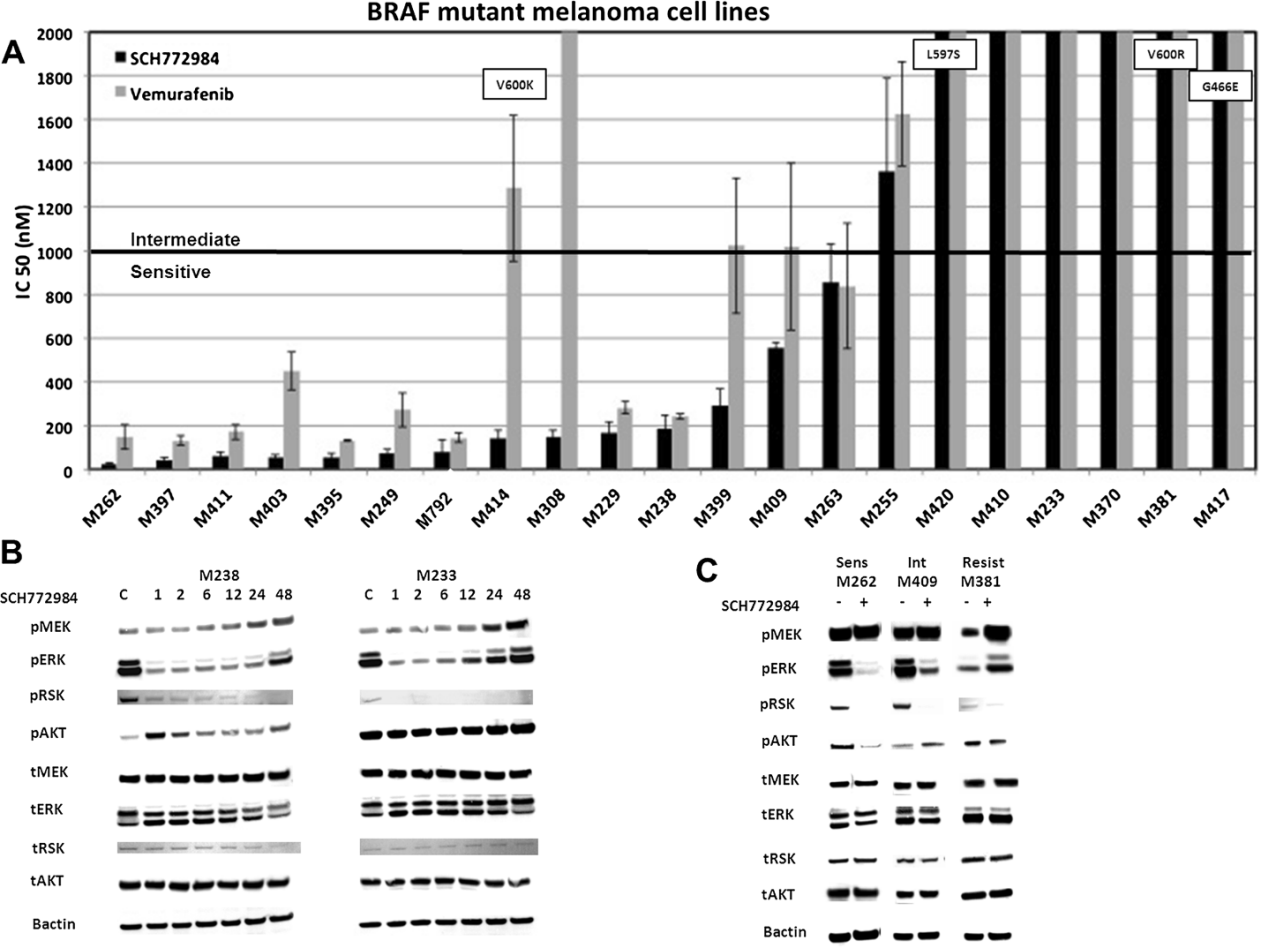
**Effect of SCH-722984 on BRAF-mutant melanoma cell lines. A**. IC_50_ (nM). 21 BRAF-mutant melanoma cell lines were exposed to 0–10 μM SCH-722984 (black bars) or vemurafenib (grey bars) and cell viability determined by ATP-based bioluminescence assay. Results are the mean of three experiments, performed in duplicates (n = 6). Error bars are standard deviation. Non-V600E substitutions are denoted in the bar graph for each corresponding cell line (M420, BRAF^L597S^; M381, BRAF^V600R^ , M417, BRAF^G466E^, M414, BRAF^V600K^). Bar at 1 μM denotes threshold between sensitive and intermediate. Resistant cell lines have IC_50_ higher than 2 μM. **B**. Timecourse effects of SCH722984 on the MAPK signaling. SCH722984-sensitive M238, SCH722984-resistant M233, were treated in a timecourse manner with 500nM SCH722984 at 1, 2, 6, 12, 24 and 48 hours compared to DMSO as solvent control **(C)**. Phosphorylated or total MEK, ERK1/2, RSK, AKT, or beta-actin as loading control were determined by western blot analysis. **C**. Effects of SCH722984 on the MAPK signaling at 24 hours. SCH722984-sensitive M262, SCH722984-resistant M381, SCH722984-intermediately sensitive M409 cells were treated for 24 h with DMSO as solvent control (-) or 500 nM SCH722984 (+).

Twenty-one melanoma cell lines containing mutations in the *BRAF* gene were evaluated to determine sensitivity to SCH772984 (ERKi). As a comparison, sensitivity to vemurafenib was also determined. *BRAF*^*V600E*^ was the most frequently observed BRAF mutation, present in 17 of 21 cell lines. M381 contains *BRAF*^*V600R*^ substitution, M414 contains *BRAF*^*V600K*^, M417 contains *BRAF*^*G466E*^ and M420 contains *BRAF*^*L597S*^ mutation. Among the 21 cell lines, sensitivity to vemurafenib or SCH-772984 fell into 3 groups: highly sensitive (50% inhibitory concentration, IC_50_ < 1 μM), intermediate sensitivity (IC_50_ 1–2 μM) and resistant (IC_50_ > 2 μM). 15 cell lines were highly sensitive to SCH-772984 with IC_50_ less than or equal to 1 μM. Of the 12 cell lines highly sensitive to vemurafenib, all contain *BRAF*^*V600E*^ and were also sensitive to SCH-772984. Interestingly, M399, M414, M308, and M409 were sensitive to SCH-772984 but only intermediately sensitive (M399, M409 and M414) or resistant (M308) to vemurafenib. With the exception of M414, all non-V600E mutants were resistant to both vemurafenib and SCH772984 (Figure 1A). As a comparison, sensitivity to the MEKi trametinib segregated all cell lines into three different groups: highly sensitive (IC_50_ < 2nM), intermediately sensitive (IC_50_ 2-30nM) and resistant (IC_50_ > 30 nM) (Additional file 1: Figure S1). In general, cell lines sensitive to SCH772984 were also sensitive to trametinib.

We next determined a time-course of SCH772984 on MAPK and PI3K/AKT pathway signaling for M238, a SCH772984-sensitive *BRAF*^*V600E*^-mutant melanoma cell line and M233, a SCH772984-resistant *BRAF*^*V600E*^-mutant melanoma cell line (Figure 1B). For both M233 and M238, treatment with 500nM SCH772984 inhibited pRSK, a known ERK1/2 downstream target, as well as pERK1/2 itself. For the resistant M233, the MAPK inhibition was strong as early as 1 hour post treatment, with decreased pERK and near-complete disappearance of pRSK. However, between 12 and 24 hours we observe a rebound in the pathway with a return to baseline pERK1/2 levels and an induction in pMEK above baseline levels by 24 hours. Little change in pAKT was seen at any timepoint up to 24 hours, though a mild induction was seen at 48 hours. For the sensitive M238, pRSK levels also decreased as early as 1 hour and levels continued to decrease thereafter. Meanwhile, pERK1/2 remained suppressed through 24 hours. By 48 hours, pERK1/2 levels increased, though at reduced levels compared to baseline. Concomitant with this, pMEK levels remained unchanged until 24 hours and increased further by 48 hours. Regarding pAKT, an early induction at 1 hour occurred, followed by decreases thereafter though never becoming completely suppressed even at 48 hours. In both cell lines, pRSK remained blocked at all timepoints, demonstrating ongoing, potent inhibition of ERK1/2 activity by SCH772984. These data supports that the distinction between sensitive and resistant cell lines could be best made based on pERK recovery at 24 hours, as recovery of the feedback loop that restores MAPK activity occurred by 24 hours in the resistant cell line whereas the sensitive cell line required longer than 24 hours. Therefore, for subsequent analyses, we selected 24 hours as the optimal timepoint to compare signaling in our cell lines.

As shown in Figure 1C, three *BRAF* mutant cell lines representative of the different sensitivity groups to SCH772984 were profiled in terms of downstream signaling inhibition at 24 hours: a highly sensitive cell line (M262, *BRAF*^*V600E*^), an intermediately sensitive cell line (M409, *BRAF*^*V600E*^), and a resistant cell line (M381, *BRAF*^*V600R*^). For M262, treatment with SCH772984 resulted in disappearance of pRSK, disappearance of pERK1/2, decrease in pAKT, and slight induction of pMEK at 24 hours. M409 had a similar cell signaling profile as M262, consistent with its modest sensitivity. For M381induction of pMEK and pERK1/2 were seen with no change in pRSK at 24 h.

To determine the effect of SCH772984 on the PI3K/AKT pathway, we first evaluated the baseline pAKT levels for a group of cell lines (Additional file 2: Figure S2). We found a weak correlation with the activity of the PI3K/AKT pathway and sensitivity to SCH772984 for BRAF mutants. For example, M238 and M409 are two clear examples of cell lines with low levels of pAKT related to sensitivity to SCH772984. For both of them, ERK inhibition with SCH772984 was accompanied by an upregulation of pAKT levels even at 24 hours treatment (Figure 1B). M233 was among the resistant *BRAF*^*V600E*^ melanoma cell lines, which appeared to have increased pAKT at baseline compared to other *BRAF* mutant cell lines. Consistent with this, M233 is a PTEN null cell line and has a concomitant *AKT1* amplification [31]. After treatment with SCH772984 (Figure 1B), these levels stay constant, indicating that dual inhibition with SCH772984 and AKT/mTOR inhibitors may be a useful strategy. In contrast, M262 is an *AKT1* amplified cell line [31] with high sensitivity to SCH772984 and vemurafenib. Treatment of M262 with SCH772984 reduced both pERK1/2 and pAKT levels, indicating blockade of the MAPK pathway and PI3K/AKT pathway at the same time (Figure 1C). In general, the presence of *AKT1* or *AKT2* amplification did not preclude sensitivity to SCH772984, as three of five such cell lines were highly sensitive to SCH772984 (M229, M249, and M262), one was intermediately sensitive (M255), and M233 and M308 were resistant (Figure 1A).

Given high baseline pAKT levels were seen in some cells resistant to ERK inhibition (Additional file 2: Figure S2) and the persistence of pAKT activity with SCH722984 treatment, we evaluated the effect of SCH772984 in combination with the AKT inhibitor MK-2206 or the mTOR inhibitor MK-8669. The addition of either the AKTi or mTORi always resulted in more potent cell growth inhibition compared to ERKi alone (Additional file 3: Figures S3A and 3B). Combining SCH772984 with the mTOR inhibitor MK-8669 was particularly synergistic. For BRAF-mutant cell line M233, both combinations resulted in more complete decrease in pERK compared to treatment with SCH772984 alone. Despite the improved inhibition of the MAPK pathway, the levels of pAKT were largely unaffected by the addition of MK-2206 or MK-8669 (Additional file 3: Figure S3C).

### Potent SCH772984-mediated ERK inhibition in *BRAF*-wild type melanoma cell lines

**Figure 2:**
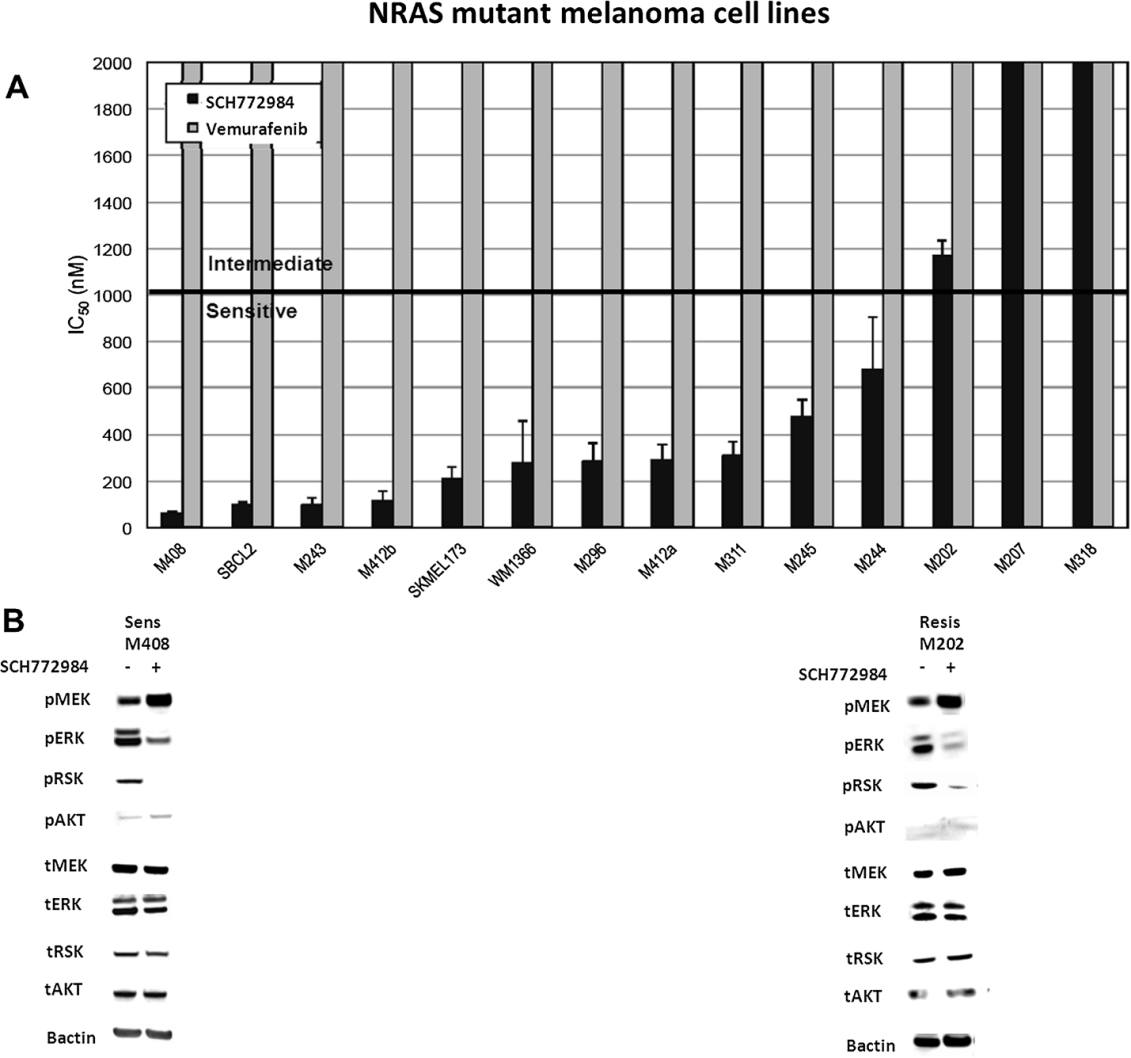
**Susceptibility of NRAS-mutant melanoma cell lines to SCH-722984. A**. IC_50_ (nM). Melanoma cell lines containing mutations in *NRAS* were exposed to 0–10 μM SCH-722984 (black bars) or vemurafenib (grey bars), and the cell viability determined. Results are the mean of three experiments, performed in duplicate (n = 6). Error bars are standard deviation. Bar at 1 μM denotes threshold between sensitive and intermediate. Resistant cell lines have IC_50_ higher than 2 μM. **B**. Effects of SCH722984 on MAPK signaling. SCH722984-resistant M202 and SCH722984-sensitive M408 were treated for 24 h with DMSO as solvent control (-) or 500 nM SCH722984 (+). Phosphorylated or total MEK, ERK 1/2, RSK, AKT or beta-actin as loading control were determined by western blot analysis.

Currently, there is no effective targeted therapy for *BRAF* wild-type melanoma, which comprises 50% of all melanomas. Fourteen *NRAS* mutant melanoma and seven cells lines with wild-type *BRA*F and *NRAS* were evaluated for SCH772984 sensitivity. As shown in Figure 2A, while all *NRAS*-mutant cell lines were resistant to vemurafenib, 11 of 14 were highly sensitive to SCH772984 (IC_50_ < 1 μM). Across the 11 NRAS sensitive cell lines, two of them were Q61L (M296 and M311), four were Q61K (M408, Sbcl2, WM1366, M245 and M244), one was Q61H (M243) and three were Q61R (SKMEL173, M296 and M412a). Interestingly, the three cell lines with IC50 > 1uM (M202, M207 and M318) were exclusively NRAS Q61L mutated. Sensitivity to trametinib are shown in Additional file 4: Figures S4A and 4B for NRAS-mutant and wild-type melanoma cell lines, respectively. Consistent with the profile for sensitive cell lines, treatment with SCH772984 for the sensitive M408 resulted in decreased pRSK, disappearance of pERK1/2, and slight induction of pMEK, with no change in total RSK, MEK, ERK 1/2, or AKT. For the resistant M202, a modest induction of pMEK with some decrease in pERK and pRSK was observed at 24 hours (Figure 2B). Treatment with SCH772984 resulted in upregulation of pAKT levels for M408 and WM1366 (Additional file 3: Figure S3C). Consistent with the synergistic growth inhibition seen with combining SCH772984 and either MK-2206 or MK-8669, pAKT levels were abrogated with the addition of MK-2206 (AKT inhibitor) and MK-8669 (mTOR inhibitor) (Additional file 3: Figure S3A-C).

**Figure 3:**
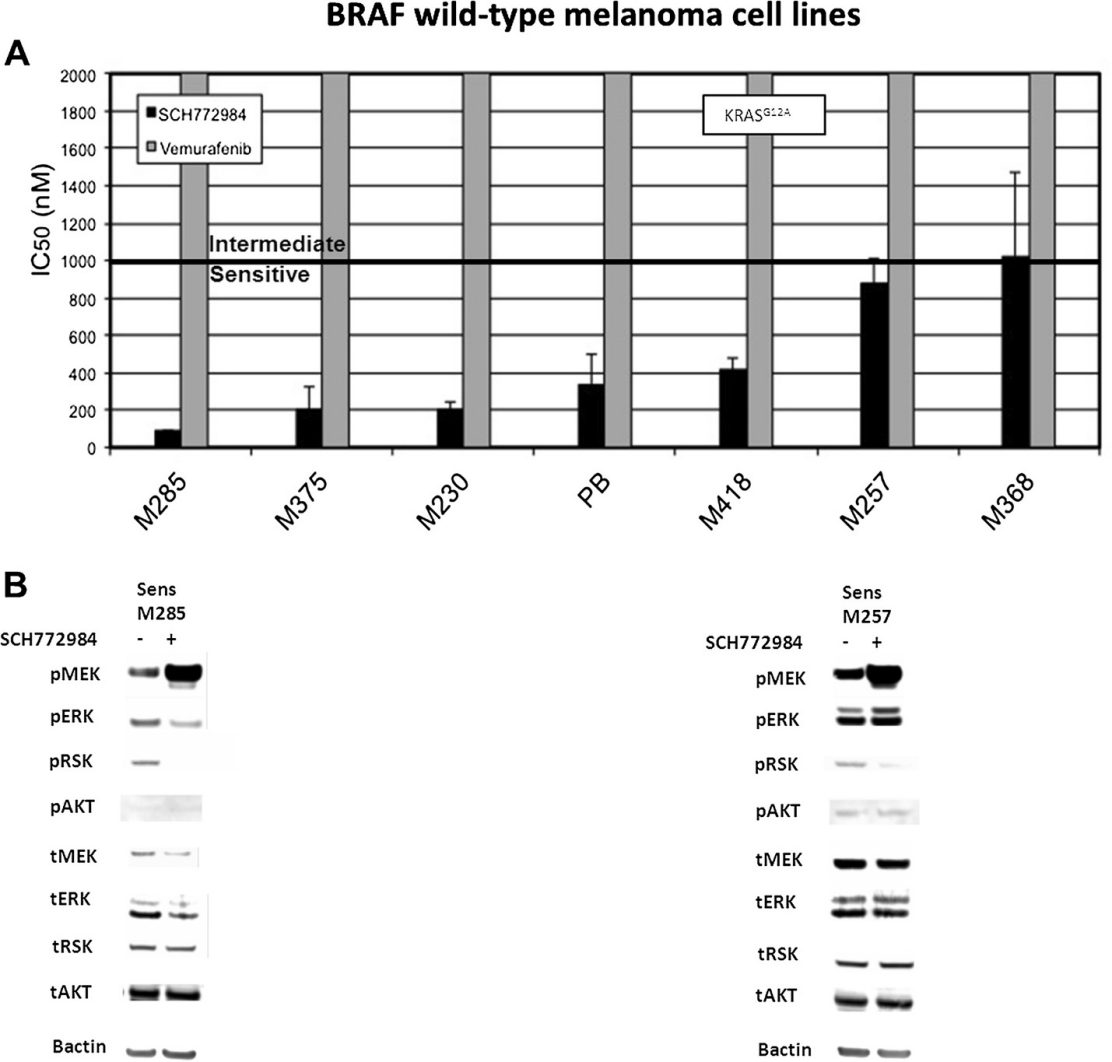
**Susceptibility of BRAF/NRAS wild-type melanoma to SCH-722984. A**. IC_50_ (nM). Cells were treated with 0–10 μM SCH-722984 (black bars) or vemurafenib (grey bars) and cell viability determined by ATP-based bioluminescence assay. Results are the mean of duplicate experiments, performed in triplicate (n = 6). Error bars are standard deviation. Bar at 1 μM denotes threshold between sensitive and intermediate. Resistant cell lines have IC_50_ higher than 2 μM. **B**. Effects of SCH722984 on MAPK signaling. SCH-722984-resistant M257 and SCH-722984-sensitive M285, were treated for 24 h with DMSO (-) or 500 nM SCH722984 (+). Phosphorylated or total MEK, ERK 1/2, RSK, AKT or beta-actin as loading control were determined by western blot analysis.

For *BRAF* and *NRAS* wild-type melanoma cell lines, all seven were sensitive to ERK inhibition, with six of seven highly sensitive to SCH772984 (Figure 3A), including M418, which is a *KRAS*^*G12A*^ mutant. Consistent with this, treatment with SCH772984 in the highly sensitive M285 line resulted in complete disappearance of pRSK, decreased pERK1/2 and induction of pMEK. M257, which is intermediately sensitive to SCH722984, had near-complete disappearance of pRSK, though pERK1/2 was slightly induced at 24 hours. Phospho-MEK was also induced in treated cells. No changes in pAKT with SCH772984 were seen in either M285 or M257 (Figure 3B). These data indicate that SCH772984 may be effective against a majority of *BRAF* wild-type cell lines including *NRAS* mutants and *BRAF/NRA*S wild-type melanoma cell lines which remain dependent on the MAPK pathway for continued growth.

### SCH722984 is effective in *BRAF*-mutant melanoma with acquired vemurafenib-resistance

**Figure 4:**
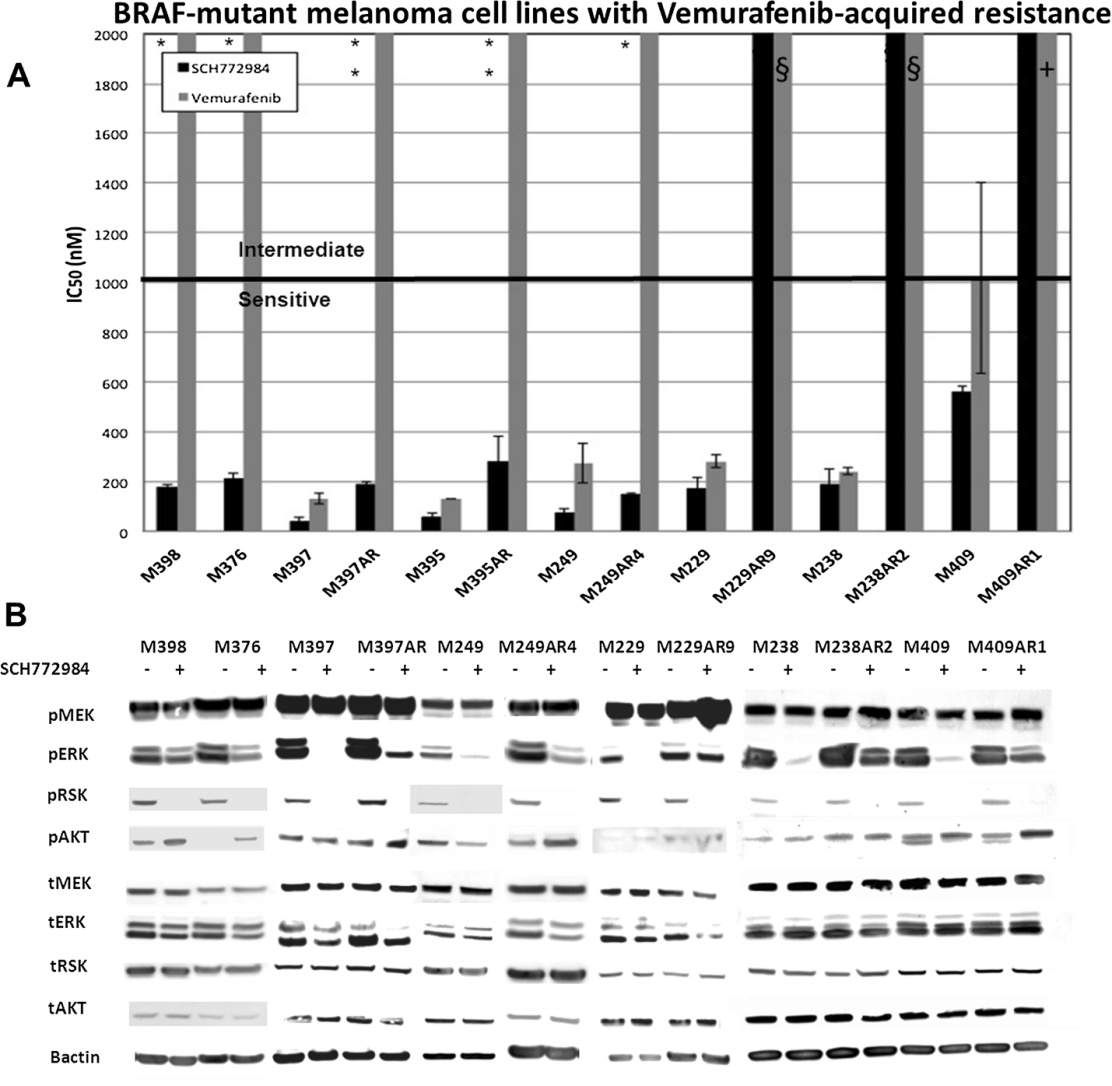
**SCH722984 in BRAF-mutant melanoma cell lines with vemurafenib-acquired resistance. A**. IC_50_ (nM) to SCH-722984 or vemurafenib. M398 and M376, two melanoma cell lines established from tumors progressing on vemurafenib, as well as six parental *BRAF* mutant melanoma cell lines and their paired *in vitro* derived vemurafenib-acquired resistance sublines (AR) were grown in the presence of 0–10 μM SCH-722984 (black bars) or vemurafenib (grey bars). Values are mean of three experiments, performed in duplicate (n = 6). Error bars are standard deviation. Bar at 1 μM denotes threshold between sensitive and intermediate. Resistant cell lines have IC_50_ higher than 2 μM. *: BRAF/NRAS double mutant, **: BRAF-splice variant, §: Receptor tyrosine kinase upreguation, +: resistance mechanism unknown. **B**. Effect of SCH722984 on MAPK signaling. Western blot analysis was performed to evaluate the effect of 24 hour exposure to 500 nM SCH&22984 (+) or DMSO (-) on phospho- or total MEK, ERK 1/2, RSK, AKT or beta-actin as loading control.

The IC_50_ of SCH722984 or vemurafenib was next determined in eight *BRAF*-mutant vemurafenib-resistant melanoma cell lines (Figure 4A). M376 and M398, two *BRAF/NRAS* double mutant cell lines derived from the same patient tumors, were highly sensitive to SCH722984, despite high resistance to vemurafenib. M376 is a spontaneously arising double mutant, whereas M398 was established from tumor from the same patient upon progression on vemurafenib. Similar to their isogenic parental cell lines, potent growth inhibition with SCH772984 was also seen for three *BRAF*^*V600E*^ mutant melanoma cell lines with *in vitro*-derived vemurafenib resistance (polyclonal population): M395AR, M397AR, and M249AR4. Previously data demonstrated that these cell lines reactivate the MAPK pathway via generation of BRAF splice variants (M395AR and M397AR) [7, 24], or via secondary *NRAS* mutation (M249AR4) [6]. In contrast, M229AR9, M238AR2 and M409AR1 remained highly resistant to SCH722984 with IC50s >2uM. For M229AR9 and M238AR2 upregulation of RTK is the mechanism of vemurafenib resistance [6]. The mechanism of resistance for M409AR is unknown at this time, and studies are ongoing to delineate the mechanism underlying BRAFi-resistance.

Consistent with the signaling for other sensitive cell lines, all three *BRAF/NRAS* double mutants (M376, M398, M249AR4) displayed decreased pRSK and pERK1/2 at 24 hours with no detectable change in pMEK. In the paired parental and acquired-resistance cell lines, treatment of the parental cell lines (M397, M249, M229, M238 and M409) with SCH722984 resulted in disappearance of pRSK, decrease in pERK1/2. Interestingly, we observed again upregulation of pAKT levels after treatment with SCH772984 in M398, M376, M397AR, M249AR4, and M409AR1, suggesting a rapid upregulation of the PI3K/AKT/mTOR pathway. This suggests that dual inhibition with SCH772984 and an AKT or mTOR inhibitor may result in more potent growth inhibition. For M397AR and M249AR4, the disappearance of pERK was robust. In contrast, for the resistant M229AR9, M238AR2 and M409AR1, though treatment with SCH772984 resulted in disappearance of pRSK, pMEK appeared to be induced and pERK1/2 remained almost unchanged (Figure 4B).

### Synergistic inhibition with combination BRAF and ERK inhibition

**Figure 5:**
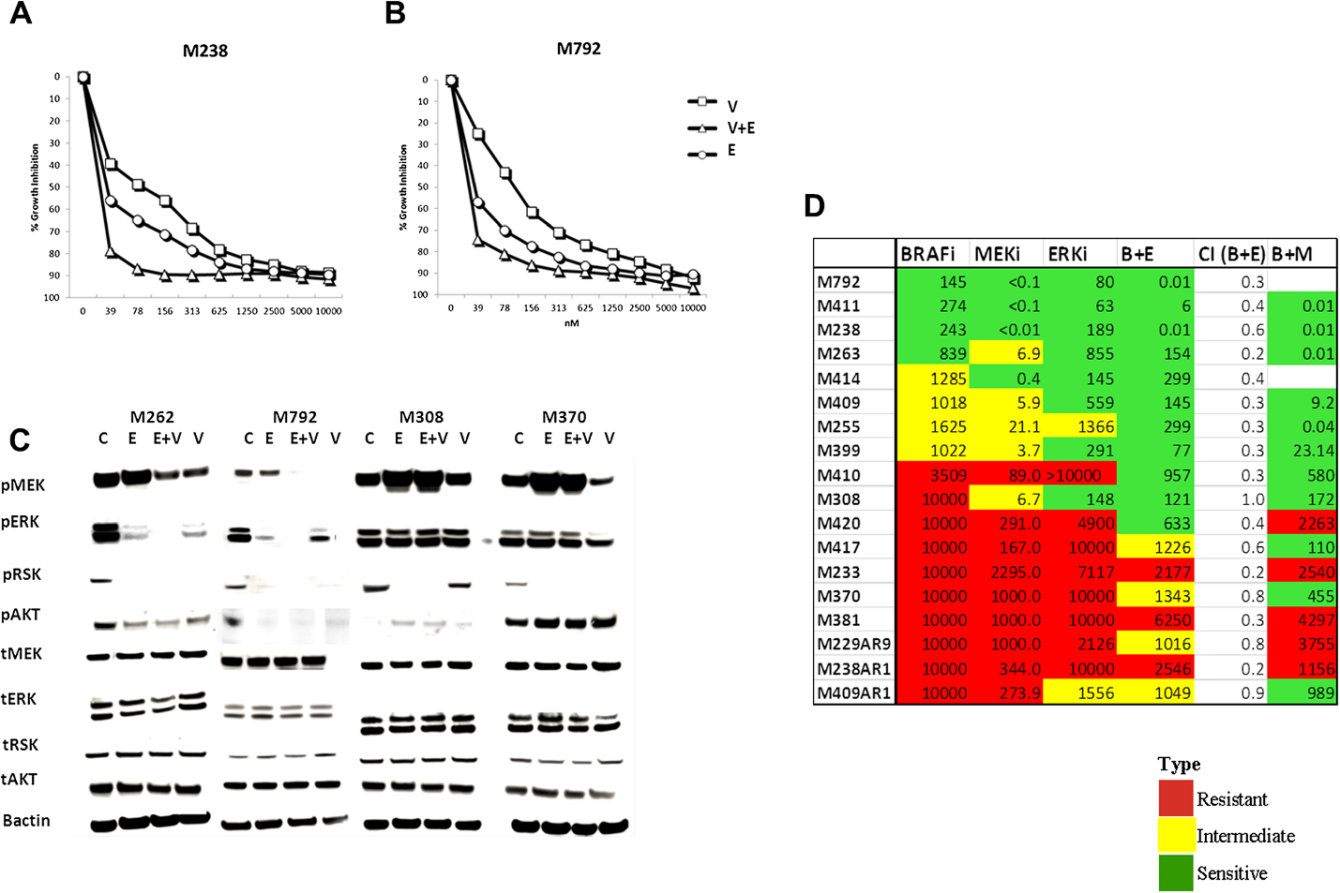
**Susceptibility of** ***BRAF*** **mutant melanoma cell lines to MAPK inhibitors.** Percent growth inhibition of **(A)** M238 and **(B)** M792. After 120 hours treatment with 0–10 μM vemurafenib (V, squares), SCH722984 (E, circles), or the combination (V + E, triangles), cell viability was determined by bioluminescence assay. Results are representative data in duplicate from three independent experiments (n = 6). **C**. Effect of BRAF-, ERK- inhibition or the combination on MAPK signaling. Cell lines were treated with DMSO (control, C), 500 nM Vemurafenib (BRAFi, B), 500 nM SCH722984 (ERKi, E) or the combination of vemurafenib and SCH722984 (B + E) for 24 hours. Western blots analyzed for phospho- and total MEK, ERK1/2, RSK, AKT and actin as loading control. **D**. IC_50_ (nM) to MAPK inhibitors and synergistic effect. BRAF-mutant melanoma cell lines were treated with 0–10 μM vemurafenib (BRAFi) or SCH722984 (ERKi), the combination (B + E) or 0–1 μM trametinib (MEKi). Green indicates sensitivity, yellow intermediately sensitive, and red for resistant. The CI column indicates the combination index of vemurafenib and SCH722984.

We next determined whether combining BRAF and ERK inhibition could result in synergistic inhibition by dual MAPK pathway inhibition. Treatment of M238 and M792, two *BRAF*^*V600E*^-mutant melanoma cell lines highly sensitive to singular treatment with vemurafenib and SCH772984, with equimolar concentrations of combined vemurafenib and SCH772984 treatment resulted in potent synergistic growth inhibition with IC_50_ of 10nM (Figure 5A, 5B). Combining vemurafenib with SCH772984 resulted in a more profound decrease in pRSK and pERK for the highly sensitive M262 and M792 lines compared to untreated controls or treatment with either agent alone. M262 resulted in decreased pAKT levels after all three treatments. For M308, which is resistant to vemurafenib and sensitive to SCH772984, as pRSK was already completely absent with treatment with SCH772984 alone, no additional effect of the combination was apparent (Figure 5C). Interestingly, at 24 hours post treatment, induction of pMEK and pERK was already seen, indicating rapid feedback recovery for M308, despite its sensitivity to SCH772984. A slight decrease in pERK1/2 was seen with SCH772984 treatment, with a compensatory increase in pMEK. The generally more resistant M308 and M370 lines, in contrast to the generally more sensitive M262 and M792 lines, did not show a decrease in pMEK when combining the two drugs, which seems to be due to the lack of activity of vemurafenib in these resistant cells. No additive effect from the dual upstream blockade could be noticed in the immediate downstream targets, indicating that the pathway remained active (Figure 5C). All cell lines sensitive to BRAFi were also sensitive to the combination. Four intermediately sensitive cell lines to BRAFi became highly sensitive to the combination, with improved pathway inhibition compared to ERK inhibition alone in all cases. More importantly, highly resistant cell lines to BRAFi became sensitive (M420, M308, M410) or intermediately sensitive (M417, M370, M229AR, M409AR1) to the combination. Three remained resistant but with a slightly improved IC_50._ C.I.s demonstrated synergy with combined BRAF and ERK inhibition for all cell lines except M308, in which dual treatment with vemurafenib and SCH772984 is only additive (Figure 5D).

We next examined whether cells inherently resistant to BRAFi could be sensitive to a MEK inhibitor (MEKi) or ERKi. Sensitivity of *BRAF* mutant melanoma cell lines to BRAFi predicted sensitivity to MEK and ERK inhibitors. Furthermore, 10 cell lines inherently resistant to BRAFi were often sensitive to MEK and ERK inhibition, and cell lines resistant to BRAFi or MEKi were relatively more sensitive to SCH722984. Indeed, combining BRAF with ERK inhibition potently decreased the equimolar IC_50_ of all cell lines and resulted in synergy in all but one case (M308), including M255, M399 and M420, which are completely resistant to individual inhibitors (Figure 5D). Because the combination on BRAF combined with MEK inhibition is currently FDA approved for *BRAF*^*V600*^ mutant melanoma, we also determined the IC_50_ for the combination of vemurafenib and trametinib. The results showed potent growth inhibition and synergy in all cell lines. Sensitivity to the vemurafenib + trametinib combination generally overlapped with sensitivity to the vemurafenib + SCH722984 combination (Figure 5D), consistent with the idea that both combinations cause dual MAPK blockade. The growth curves for the vemurafenib + trametinib combinations are shown in Additional file 5: Figure S5.

### Effects of MAPK pathway inhibition on cell cycle progression and apoptosis

**Figure 6:**
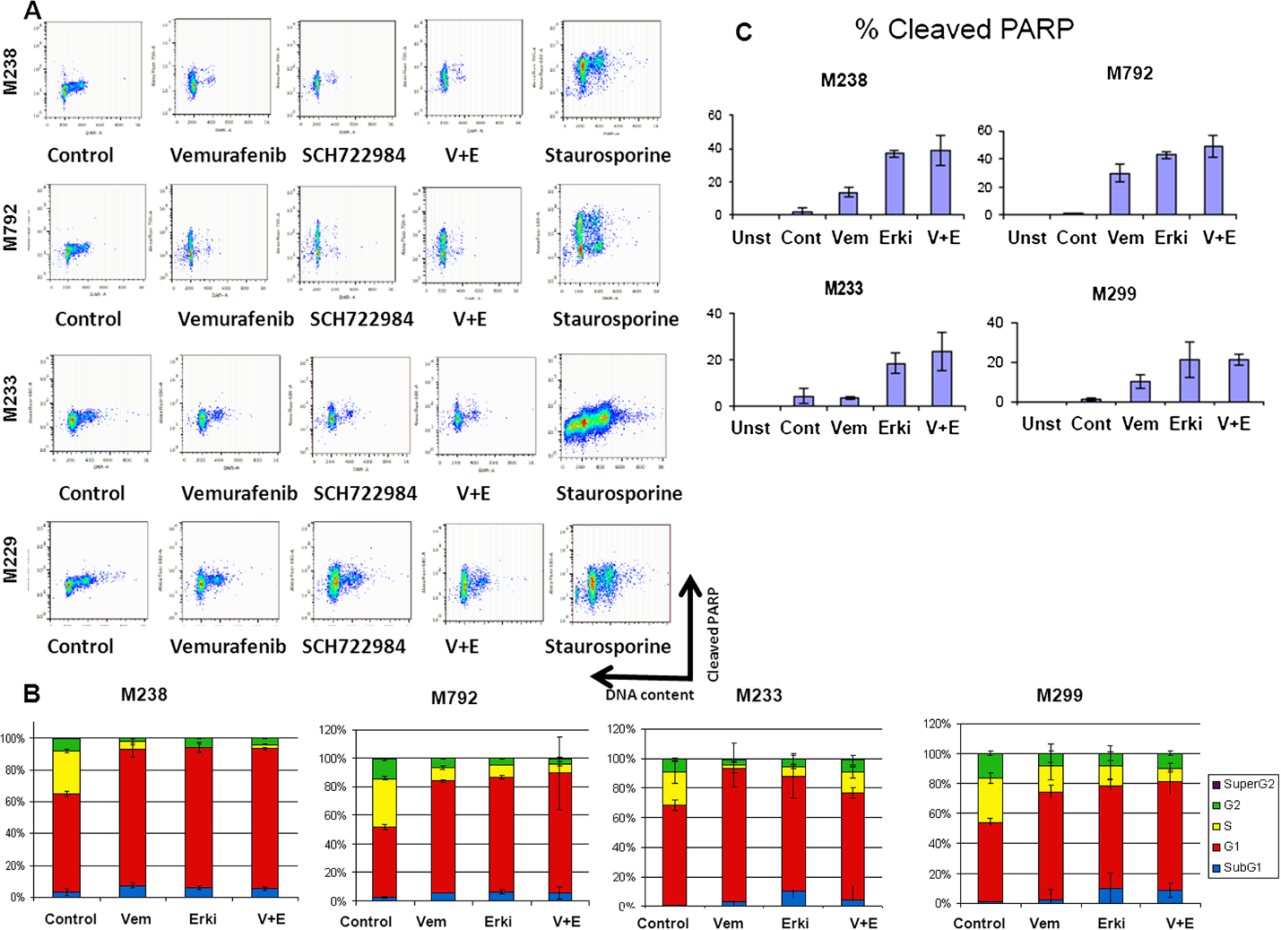
**Effect of SCH722984, vemurafenib or the combination on cell cycle progression and apoptosis in BRAF-mutant melanoma cell lines.** Two sensitive cell lines (M238 and M792) and two resistant cell lines (M233 and M299) were exposed to DMSO as vehicle control (Unst, control unstained: Control, control stained), 1 μM vemurafenib (Vem), SCH722984 (ERKi), the combination (V + E) or 1 μM staurosporine for 48 hours. **A**. Cell cycle progression was tested by DAPI staining solution and induced apoptosis by cleaved PARP (PARP-Ax700). Figures are representative of triplicate experiments. **B**. Quantitative analysis of the cell cycle progression by DAPI staining using flow cytometry shows the percentage of cells in sub-G0 (blue), G0/G1 (red), S phase (yellow), or G2/M (green). Numbers on the bar graph represent percentage of cells in G0/G1. Columns represent mean values of three independent experiments (n = 3); bars, SEM. **C**. Apoptosis in response to MAPK inhibitors. Percentage of apoptotic cells positive for cleaved PARP (PARP-Ax700) in this four melanoma cell lines. Columns represent mean values of three independent experiments (n = 3); bars, SEM; *, P < 0.05.

To determine the effect of BRAF or ERK inhibition on cell cycle progression and apoptosis, cells were treated with SCH772984, alone or in combination with vemurafenib for 48 hours then stained with DAPI and intracellularly for cleaved PARP and analyzed by flow cytometry. Treatment with either of these two inhibitors resulted in an increase in the sub-G0 population, the G1 population, as well as an increase in cleaved PARP levels which indicates apoptotic cells (Figure 6A). A modest increase of 3-10% was seen in the sub-G0 population for all cell lines treated with vemurafenib, SCH772984 or the combination. The amount of subG1 increase did not correlate with sensitivity or resistance to the MAPK pathway inhibitors. In contrast, for the G0-G1 populations, there was a correlation with sensitivity, with an up to 40% increase in G0-G1 population seen in the sensitive cell lines M238 and M792, while the resistant cell lines M233 and M299 demonstrated only a 10% increase in the G0-G1 population for the combination treatment. Concomitant with the increase in G0-G1, a decreased proportion of cells were observed in S-phase, with the largest decreases of over 20% seen in the sensitive cell lines M792 and M238 (Figure 6B). Treatment of the sensitive cell lines, M238 and M792, with SCH772984 alone or in combination with vemurafenib resulted in a dramatic increase in cleaved PARP reaching induced percentages around 40-50%. In comparison, the resistant cell lines (M233 and M299) had cleaved PARP at 20-25% (Figure 6C). With the exception of M299, a statistically significant increase in cleaved PARP was seen in all cell lines treated with SCH772984, or the combination of SCH772984 and vemurafenib, compared to vemurafenib alone. Combinatorial treatment offered a statistically significant increase in cleaved PARP compared to vemurafenib alone in all cell lines (20% increase in sensitive cell lines and 10% increase in resistant cell lines compared to vemurafenib alone). However only a trend towards increased cleaved PARP fractions was observed comparing combinatorial treatment with SCH772984 alone without reaching statistical significance.

To address whether there are differences in MEK inhibition or ERK inhibition on apoptosis, we performed cell cycle analysis on three melanoma cell lines with distinct sensitivity profile to SCH772984: M263 (sensitive), M255 (intermediately sensitive) and M370 (resistant). All three cell lines demonstrated good synergy for the combination of vemurafenib and SCH772984. Cell lines treated with SCH772984 or the combination of vemurafenib + SCH772984 had the highest levels of cleaved PARP. In comparison, trametinib, did not induce the same levels of apoptosis in any case. In terms of effects on the cell cycle, G0-G1 arrest was maximally induced by ERK inhibition also (Additional file 6: Figure S6). This data support the potent activity of SCH772984 both as a single agent and in combination.

### Combining BRAF and ERK inhibition delays the development of resistance

**Figure 7:**
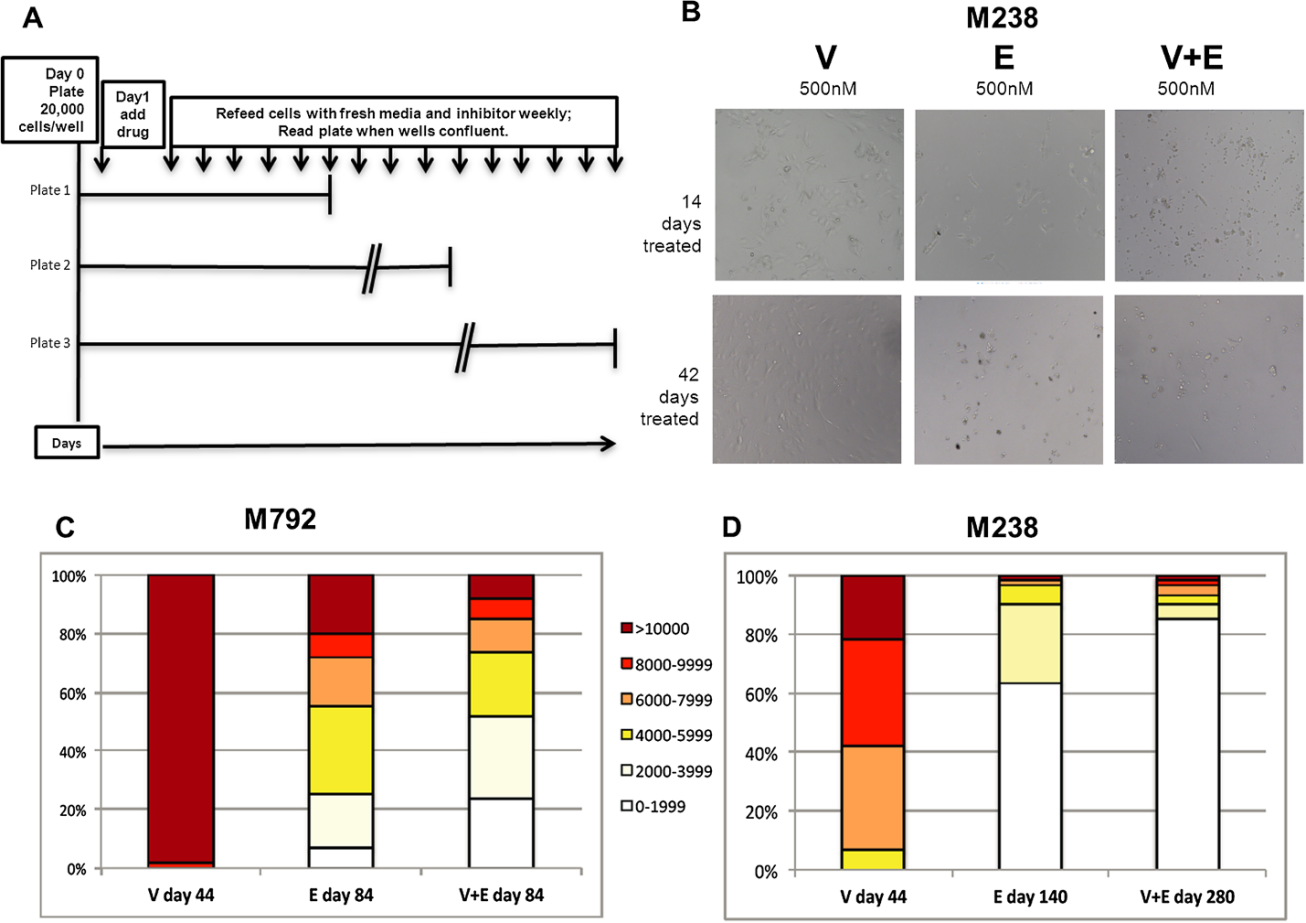
**Effect of combined MAPK inhibition on long-term melanoma cultures.** M238 and M792, two MAPK inhibitor-sensitive cell lines, were chronically exposed to 500nM of Vemurafenib (V), SCH722984 (E) or combination (V + E) in 96-well plates. **A**. Schematic. For each cell line, 20,000 cells/well were plated on 96-well plates. The following day, all cells on the plate were treated with 500nM of each drug or combination. Fresh media containing the appropriate inhibitor(s) was added weekly. When cells in several wells were confluent, viable cells were quantified by MTS assay and cell numbers were determined by comparing with a standard curve of known cell numbers. **B**. Microscopic imaging of treated M238 cells photographed at 14 days (top) or 44 days (bottom) of treatment. **C**. Quantitative analysis of viable cells for M792 at 44 days (V) or 84 days (E and V + E). **D**. Quantitative analysis of viable cells for M238 at 44 days (V), 140 days (E) or 280 days (V + E). The percentage of wells with 1–1,000 (white), 2,000-3,999 (tan), 4,000-5,999 (yellow), 6,000-7,999 (orange), 8,000-9,999 (red) and >10,000 (brown) viable cells are shown. Data are representative of triplicate independent experiments.

Given the potent synergistic inhibition seen when combining vemurafenib and SCH772984, we hypothesized that chronic exposure to dual inhibition would significantly delay the development of resistance compared to either single agent alone. To test this hypothesis, long term cultures were established in 96-well plates to allow both qualitative and quantitative assessment of the effect of each drug alone or their combination on M238 and M792, which are highly sensitive to both inhibitors and for which dual therapy was highly synergistic (Figure 7A). Treatment with 500nM vemurafenib initially suppressed growth of M238 and M792, however, by 42 days of treatment, several wells were confluent and cells had normal morphology, indicating development of BRAFi-resistance. In contrast, very few cells were seen in plates treated with ERKi alone or in combination at 42 days (Figure 7B). For ERKi-treated M792 plates, confluent wells were not seen until 84 days. When cell viability was quantified at day 44 for BRAFi-treated cells, over 90% of the wells had >10,000 viable cells. In contrast, many fewer viable cells were seen for M792 treated with ERKi alone or in combination with BRAFi measured at day 84. Only 20% of the wells treated with ERKi alone and <5% of wells treated with the combination had >10,000 cells (Figure 7C). For M238, similar data were seen, though in this case, treatment with 500nM ERKi alone delayed the development of resistance until 140 days. Most strikingly, for M238, 280 days, twice that for SCH772984 alone, were required for resistance to the combination of vemurafenib and SCH772984 to develop (Figure 7D). Taken together, these data provide preliminary *in vitro* evidence that SCH772984 may more potently and more durably inhibit *BRAF*-mutant melanoma compared to single agent vemurafenib, and that the combination of BRAF and ERK inhibition results in better inhibition than either alone. These data are intriguing and should be further validated *in vivo*.

## Discussion

Our work reports the potent activity of SCH772984 against a large panel of well-characterized melanoma cell lines. SCH772984 is highly active amongst *BRAF-*mutant, *NRAS-*mutant, double mutant and double wild-type melanoma cell lines, and this drug potently inhibited some lines with innate resistance or acquired resistance to vemurafenib. The efficacy of SCH772984 in *NRAS*-mutant and double wild-type melanoma was also striking with the majority being highly sensitive.

Previously published reports have documented promising preclinical activity of ERK inhibition in BRAFi or MEKi resistant cell line models [30, 32, 33]. Our data are consistent with the work of Carlino *et al*., who demonstrated that SCH772984 induced cell death for all sensitive cell lines. SCH772984 also resulted in growth inhibition of only melanoma cell lines with MAPK-dependent mechanisms of BRAFi-resistance, and not those with RTK upregulation as the mechanism of BRAFi resistance. Furthermore, their work demonstrated that trametinib less completely inhibited MAPK signaling compared to inhibition with VX-11e, an ERKi tool compound [33]. Since resistance to BRAFi or MEKi is highly dominated by the reactivation of the MAPK pathway (acquisition of *RAS* mutations, *BRAF*^*V600*^ amplifications, *MEK* mutations, *BRAF*^*V600E*^ amplification or the Δ2-8 BRAF splice variant [30] or secondary RTK overexpression), SCH772984 is a promising strategy to delay or revert resistance and has demonstrated activity in our BRAFi-resistant melanoma cell lines with reactivation of the MAPK pathway mechanism of resistance. Unfortunately resistance to SCH772984 may appear and resistance mechanisms will likely differ from those characterized thus far. We observed that increased pAKT levels at baseline levels were tightly correlated with intrinsic resistance to SCH772984 in the BRAF- mutant melanoma cell lines. Little to no pAKT activity was found in two NRAS mutant or two double wild type cell lines. Indeed, Carlino *et al*. hypothesize consistently in their work that pAKT levels for BRAF mutant melanoma cells may play a key role in resistance. SCH772984 is unique, having both ATP-competitive and non-competitive properties, leading to suppression of both the downstream factors of and pERK1/2 itself which was observed in short term treatment (less than 12 hours) of cell lines tested in this study. This was further elucidated by Morris *et al*., who demonstrated that the inhibition of pERK1/2 is not just mediated by inhibitor binding to MEK. Instead, they hypothesize that binding of SCH772984 to ERK protein not only prevents ATP binding and therefore phosphorylation of downstream targets like RSK, but that ERK binding also inhibits the ability of MEK protein to access and phosphorylate ERK1 at threonine 202/tyrosine 204 and ERK2 at threonine 185/tyrosine 187 [30]. Additionally, the data herein underscore the importance of MAPK feedback loops in sensitivity to SCH772984 given the consistent finding that cell lines resistant to SCH772984 are able to rapidly recover pMEK and pERK activity, while those sensitive to SCH772984 have delayed ability to do so. One notable exception to this signaling pattern is M308, which demonstrated a rapid recovery of MAPK activity even at 24 hours after ERKi-treatment, despite its sensitivity to SCH772984. In work ongoing in the lab, among 5 BRAF-mutant cell lines sensitive to SCH772984, M308 was the only cell line to develop resistant subclones after 2 months of chronic exposure to SCH772984 (data not shown). This indicates that feedback recovery may be an important predictor of development of acquired resistance to ERKi. Given that pRSK remains suppressed at all time points tested, reactivation of the MAPK pathway may allow signaling through other ERK downstream targets, such as cyclin D1.

Previous data supports that pERK signaling recovers rapidly after vemurafenib treatment, yet cells remain growth arrested [16]. Besides the synergistic effect of vemurafenib and SCH772984, our data supports a strong induction of apoptosis with the ERKi compared to the BRAFi alone. This should encourage combined therapy approaches to strengthen the therapy against feedback loops reactivated after single point blockade. The strategic aspect of ERK inhibition both blocking downstream in the MAPK pathway and blocking the cross-talk with Pi3K/AKT can support a rationale to combine inhibition of both pathways.

Both the non-competitive inhibitory properties of SCH772984 and its ability to cause reactivation of the MAPK pathway may explain the synergy seen with combination BRAF- and ERK-inhibition in the vast majority of *BRAF*-mutant melanoma. It may also explain the dramatic delay in development of resistance seen with BRAF-inhibitor in *BRAF*-mutant melanoma tested in this study. At first glance, the idea of inhibiting two points in the same pathway may seem redundant. However, as shown herein, the dual inhibition approach may be a good strategy to control the feedback loop reactivation that occurs with singular treatment with ERKi. Carlino *et al*. [33] , recently published that combinatorial blockade with relatively high concentrations of the ERKi, Vx-11e (10 μM) and PI3K/AKT inhibitor BEZ235 (2 μM) more completely induced cell death in BRAF-inhibitor resistance melanoma cell lines compared to the combination of trametinib (10nM) and BEZ235. Consistent with this, Lassen *et al.* recently demonstrated that MAPK and PI3K pathway dual inhibition can effectively inhibit melanoma cells [34]. Given the likely differences in toxicity profile of both combinatorial approaches, clinical evaluation of both BRAFi/ERKi and ERKi/PI3Ki should be pursued. Further investigations regarding the mechanisms underlying both primary and acquired resistance to SCH772984 are ongoing.

## Conclusions

Currently, at least two ERK inhibitors are in phase I studies, including MK8353, a clinical grade analog of SCH772984, and BVD-523. Our preclinical findings demonstrate the ability of SCH772984 to inhibit the majority of melanoma cell lines tested. Over 50% of all melanoma patients are non-*BRAF* mutated, with *NRAS*-mutant and double wild-type comprising 15-20% and 40% of melanoma patients, respectively. As there are currently no FDA-approved targeted therapies for these non-*BRAF* mutated melanoma, these data provide intriguing pre-clinical basis for further development and testing of ERK inhibitors for these melanoma subtypes, as well as for combinatorial therapy with BRAF and ERK inhibitors in BRAF-mutant melanoma. Therefore, ERK inhibitors hold much promise to augment the armamentarium of effective targeted therapies for melanoma, regardless of *BRAF* mutational status and irrespective of sensitivity to BRAF inhibitors.

## Materials and methods

### Reagents and cell lines

SCH722984 (Erk inhibitor), MK-2206 (AKT inhibitor) and MK-8669 (mTOR inhibitor) were obtained through a materials transfer agreement with Merck Sharp & Dohme Corp. (Whitehouse Station, New Jersey). Vemurafenib and trametinib were commercially purchased (Selleck Chemicals, Houston, TX). All drugs were reconstituted in 100% dimethyl sulfoxide (DMSO) to a final concentration of 10 mM. With the exception of Sbcl2, SKMel173, WM1366, which were obtained from Dr. Roger S. Lo (UCLA), all other human melanoma cell lines were established from patient biopsies under UCLA- IRB #02–08-067, as previously described [31]. Cells were cultured in RPMI 1640 with L-glutamine (Mediatech, Inc, Manassas, VA), 10% fetal bovine serum (FBS, Omega Scientific, Tarzana, CA), and 1% penicillin, streptomycin and fungizone (PSF, Omega Scientific). Cultures were incubated in a water-saturated incubator at 37°C with 5% CO_2_.

### Cell viability

All cell lines were treated in duplicates with 0–10 μM of SCH722984, vemurafenib and trametinib alone or in combination and constant amount of DMSO for all the conditions. After incubation for 72–120 hours, the cell viability was determined using CellTiter-Glo Luminescent Cell Viability Assay (Promega, Madison, WI), an ATP-based bioluminescent assay, as per manufacturer’s instructions. Each experiment was repeated independently at least 3 times.

For long-term culture experiments, M238 and M792 cell lines were plated at 20,000 cells per well in 96-well plates and chronically exposed to 500nM of vemurafenib, SCH722984 or the combination. Cells were re-fed with fresh media containing the appropriate inhibitor(s) weekly. Pictures were taken weekly to document visual differences in between conditions. Plates were considered for cell viability reading with [3-(4,5-dimethylthiazol-2-yl)-5-(3-carboxymethoxyphenyl)-2-(4-sulfophenyl)-2H-tetrazolium (MTS) (Promega) as previously described [31] when several wells appeared visually to be >90% confluent. The number of viable cells in each well was determined based on a standard curve of known cell numbers. Each condition was repeated in triplicate independent experiments.

### Western blotting

All melanoma cells were washed with ice-cold phosphate buffered saline (PBS) twice and lysed with RIPA buffer containing phosphatase and protease inhibitors (all from Sigma Aldrich, St. Louis, MO). Protein extracts were separated with SDS-PAGE in 4-12% tris-glycine gels and transferred to immun-blot PVDF membrane. After blocking for 1 hour in PBS containing 0.1% Tween 20 and 5% nonfat milk or 5% bovine serum albumin (BSA) in PBS, the membrane was exposed to various primary antibodies overnight, followed by secondary antibodies conjugated to horseradish peroxidase. ECL-Plus kit (Amersham Biosciences Co, Piscataway, NJ) was used to check immunoreactivity and blots were scanned using a Typhoon scanner (Amersham Biosciences Co.). Primary antibodies included pERK Thr202/Tyr204, total ERK, pMEK Ser217/221, total MEK, pAKT Ser473, total AKT, pRSK, total RSK, beta-actin (all from Cell Signaling Technology, Danvers, MA).

### Cell-cycle analysis and assessment of apoptosis by flow cytometry

M238, M792, M299 and M233 melanoma cell lines were incubated with 1 μM of DMSO as vehicle control, vemurafenib, SCH772984, or the combination for 48 hours. Cells were collected and fixed with BD Cytofix/Cytoperm (BD Biosciences, San Jose, CA), washed with BD Perm/Wash Buffer 1x and resuspended in 500 μL of 4,6-diamidino-2-phenylindole (DAPI) solution at a final concentration of 2 mg/mL, 0.001% Nonident P-40, and 1% BSA in PBS (Sigma-Aldrich). Analysis was performed after acquiring 12,000 cellular events in G0-G1 gate per sample. For analysis of apoptosis, cells were checked at the level of cleaved poly [ADP-ribose] polymerase (PARP). After fixation and permeabilization as described above, cells were stained with anti–PARP-Alexafluor700 antibody (clone F21-852; BD Biosciences). A minimum of 12,000 cellular events per sample were collected by flow cytometry. All flow cytometry experiments were carried out using an LSRII (BD Biosciences), using biexponential axes. Data were analyzed using FlowJo (Tree Star, Inc, Ashland, OR).

### Statistical analysis

Data analysis was performed with Microsoft Excel and GraphPad Prism to determine the 50% inhibition concentration (IC_50_). Synergistic, additive or antagonistic effects of drug combinations were determined using Calcusyn software (version 2.0, Biosoft, Cambridge, United Kingdom) to determine the combination indices (C.I.) as previously described [35], where synergy is defined as C.I. <1, additivity C.I. ≃ 1, and antagonism is C.I. >1.

## Electronic supplementary material

Additional file 1: Figure S1: Effect of trametinib on *BRAF* mutant melanoma cell lines. IC_50_ (nM). Ninteen *BRAF* mutant melanoma cell lines were exposed to 0–1 μM trametinib and cell viability determined by ATP-based bioluminescence assay (CellTiter-Glo, Promega). Results represent mean of duplicate assay performed in three independent experiments. (PDF 47 KB)

Additional file 2: Figure S2: Levels of pAKT across a group of BRAF, NRAS and double wild-type melanoma cell lines. 8 BRAF-mutant, 2 NRAS-mutant and 2 wild type melanoma cell lines in order of increasing IC_50_ sensitivities to SCH772984 were evaluated by Western Blot analysis for baseline pAKT levels. (PDF 31 KB)

Additional file 3: Figure S3: Effects of SCH722984 combined with MK-2206 (AKT inhibitor) and MK-8669 (mTOR inhibitor) in BRAF mutant cell lines. A. IC_50_s of SCH722984 alone or in combination with MK-2206 or MK-8669. After 120 hours treatment with 0–10 μM SCH722984, SCH722984+ MK-2206 or SCH722984+ MK-8669, cell viability was determined by bioluminescence assay. Results are representative data in duplicate from three independent experiments (n = 6). B. Percent growth inhibition for two BRAF-mutant melanoma cell lines (M233 and M411) and two NRAS-mutant cell lines (M409 and WM1366). After 120 hours treatment with 0–10 μM SCH772984 + MK2206 (ERKi + AKTi, squares), SCH722984 + MK-8669 (ERKi + mTORi, triangles), or the SCH772984 (ERKi, circles), cell viability was determined by bioluminescence assay. Results are representative data in duplicate from three independent experiments (n = 6). C. Effect ERK- inhibition alone or the combination with AKT/mTOR inhibitors on MAPK signaling. Cell lines were treated with DMSO (control, C), 1 uM SCH722984 (ERKi, E) or the combination of SCH722984+ MK-2206 and SCH722984+ MK-8669 at 1 uM for 24 hours. Western blots analyzed for phospho- and total ERK1/2, AKT and actin as loading control. (PDF 78 KB)

Additional file 4: Figure S4: Effect of trametinib on NRAS mutant and double wild type cell lines. IC50 (nM). 14 NRAS mutant and 7 double wild-type melanoma cell lines were exposed to 0-1 μM trametinib and cell viability was determined by ATP-based bioluminescence assay (CellTiter-Glo, Promega). Results represent mean of duplicate assay performed in three independent experiments. (PDF 40 KB)

Additional file 5: Figure S5: Combinatorial effect of Vemurafenib with SCH772984 or Trametinib. Percent growth inhibition of BRAF mutant cell lines. After 120 hours treatment with 0–10 μM vemurafenib (squares) combined with 0-10 uM SCH722984 (circles), or 0–10 μM vemurafenib combined with 0-1 μM trametinib, cell viability was determined by bioluminescence assay. Results are representative data in duplicate from two independent experiments (n = 6). (PDF 348 KB)

Additional file 6: Figure S6: Effect of SCH722984, vemurafenib or the combination on cell cycle progression and apoptosis in BRAF-mutant melanoma cell lines. A sensitive cell line (M263), intermediate sensitivity (M255) and resistant to SCH722984 (M370) were exposed to DMSO as vehicle control (ControL), 1 μM vemurafenib (Vemurafenib), SCH722984 (ERKi), 50nM trametinib (Trametinib), the combination of 1 μM vemurafenib + 1 μM SCH722984 (V + E) or the combination of 1μMvemurafenib + 50nM trametinib (V + T) for 48 hours. A. Cell cycle progression example for M255 was tested by DAPI staining solution and induced apoptosis by cleaved PARP (PARP-Ax700). B. Apoptosis in response to MAPK inhibitors. Percentage of apoptotic cells positive for cleaved PARP (PARP-Ax700) in this three melanoma cell lines. B. Quantitative analysis of the cell cycle progression by DAPI staining using flow cytometry shows the percentage of cells in sub-G0, G0/G1, S phase, or G2/M. (PDF 199 KB)

## Abbreviations

AR: Acquired resistant
C.I.: Combination Index
DAPI: 4,6-diamidino-2-phenylindole
DMSO: Dimethyl sulfoxide
FBS: Fetal bovine serum
IC_50_: 50% inhibition concentration
IGF1R: Insulin growth factor receptor 1
MTS: [3-(4,5-dimethylthiazol-2-yl)-5-(3-carboxymethoxyphenyl)-2-(4-sulfophenyl)-2H-tetrazolium
μM: Micromolar
nM: Nanomolar
PARP: Poly [ADP-ribose] polymerase
PBS: Phosphate buffered saline
PDGFRβ: Platelet-derived growth factor beta
PSF: Penicillin, streptozocin, fungizone
RTK: Receptor tyrosine kinase.
