# Erratum to: Antitumor activity of the ERK inhibitor SCH722984 against BRAF mutant, NRAS mutant and wild-type melanoma

**DOI:** 10.1186/s12943-015-0393-2

**Published:** 2015-07-02

**Authors:** Deborah JL Wong, Lidia Robert, Mohammad S Atefi, Amanda Lassen, Geetha Avarappatt, Michael Cerniglia, Earl Avramis, Jennifer Tsoi, David Foulad, Thomas G Graeber, Begonya Comin-Anduix, Ahmed Samatar, Roger S Lo, Antoni Ribas

**Affiliations:** Department of Medicine, Division of Hematology-Oncology, University of California Los Angeles (UCLA), 11-934 Factor Building, Los Angeles, CA USA; Department of Molecular and Medical Pharmacology, UCLA, Los Angeles, CA USA; Department of Surgery, Division of Surgical-Oncology, UCLA, Los Angeles, CA USA; Jonsson Comprehensive Cancer Center at UCLA, 10833 Le Conte Avenue, Los Angeles, CA 90095-1782 USA; Discovery Oncology Merck Research Laboratories, Merck Research Laboratories, Boston, Massachusetts USA; Department of Medicine, Division of Dermatology, UCLA, Los Angeles, California USA; University of Applied Sciences, Vienna, Austria; Universitat Autonoma de Barcelona, UAB, Barcelona, Spain

## Correction

After publication of this work [1], we noted that we repeatedly had a typo mistake across the manuscript. The compound we used to test our cell lines was SCH772984 and not SCH722984. The typo was found misspelled in 54 occasions, including the title.

We also wanted to add an affiliation detail to the author’s list, as noted above.

We regret the mistake and upload this note to clarify it.
