# Supplementary figures and images for: Antitumor activity of the ERK inhibitor SCH722984 against BRAF mutant, NRAS mutant and wild-type melanoma
Source: Mol Cancer. 2014 Aug 20;13:194. doi: 10.1186/1476-4598-13-194 (PMC4155088; doi:10.1186/1476-4598-13-194)

Supplemental Figure 1

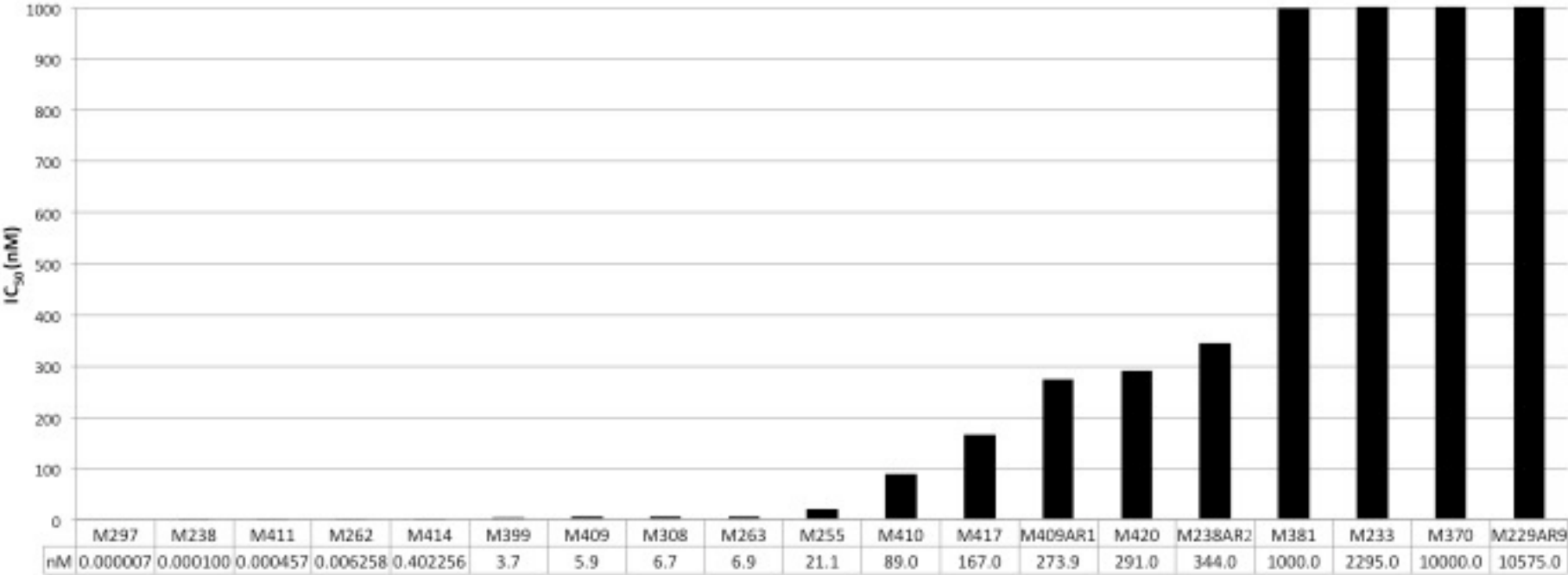

Supplement: Supplementary file 1 — Additional file 1: Figure S1: Effect of trametinib on BRAF mutant melanoma cell lines. IC50 (nM). Ninteen BRAF mutant melanoma cell lines were exposed to 0–1 μM trametinib and cell viability determined by ATP-based bioluminescence assay (CellTiter-Glo, Promega). Results represent mean of duplicate assay performed in three independent experiments. (PDF 47 KB) [file 12943_2014_1396_MOESM1_ESM.pdf]

Supplemental Figure 2

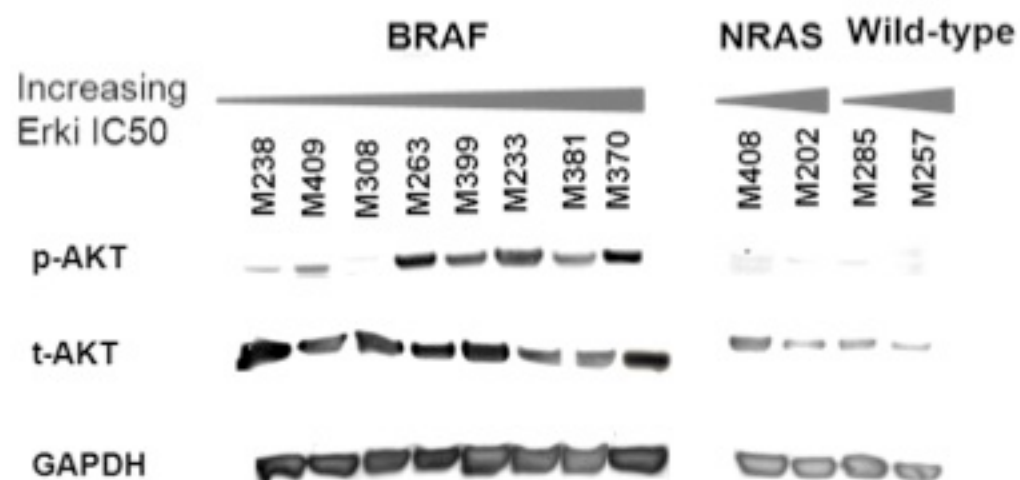

Supplement: Supplementary file 2 — Additional file 2: Figure S2: Levels of pAKT across a group of BRAF, NRAS and double wild-type melanoma cell lines. 8 BRAF-mutant, 2 NRAS-mutant and 2 wild type melanoma cell lines in order of increasing IC50 sensitivities to SCH772984 were evaluated by Western Blot analysis for baseline pAKT levels. (PDF 31 KB) [file 12943_2014_1396_MOESM2_ESM.pdf]

# Supplemental Figure 4

**A**

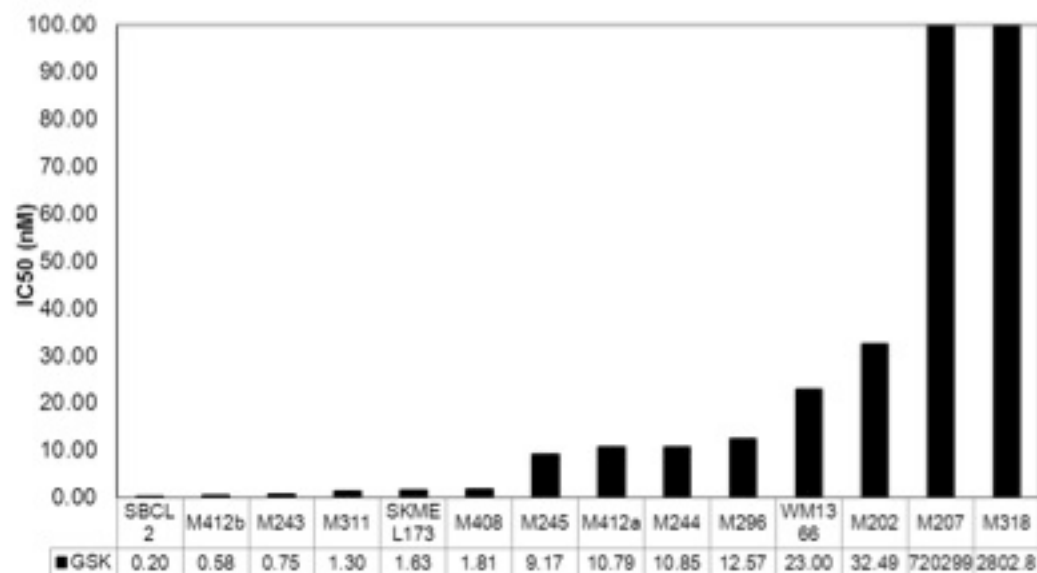

**B**

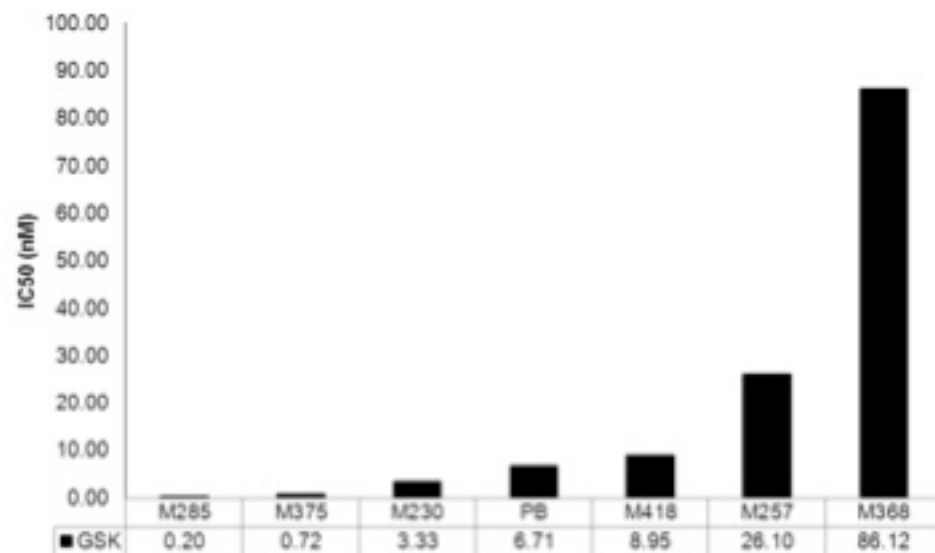

Supplement: Supplementary file 4 — Additional file 4: Figure S4: Effect of trametinib on NRAS mutant and double wild type cell lines. IC50 (nM). 14 NRAS mutant and 7 double wild-type melanoma cell lines were exposed to 0-1 μM trametinib and cell viability was determined by ATP-based bioluminescence assay (CellTiter-Glo, Promega). Results represent mean of duplicate assay performed in three independent experiments. (PDF 40 KB) [file 12943_2014_1396_MOESM4_ESM.pdf]

Supplemental Figure 5

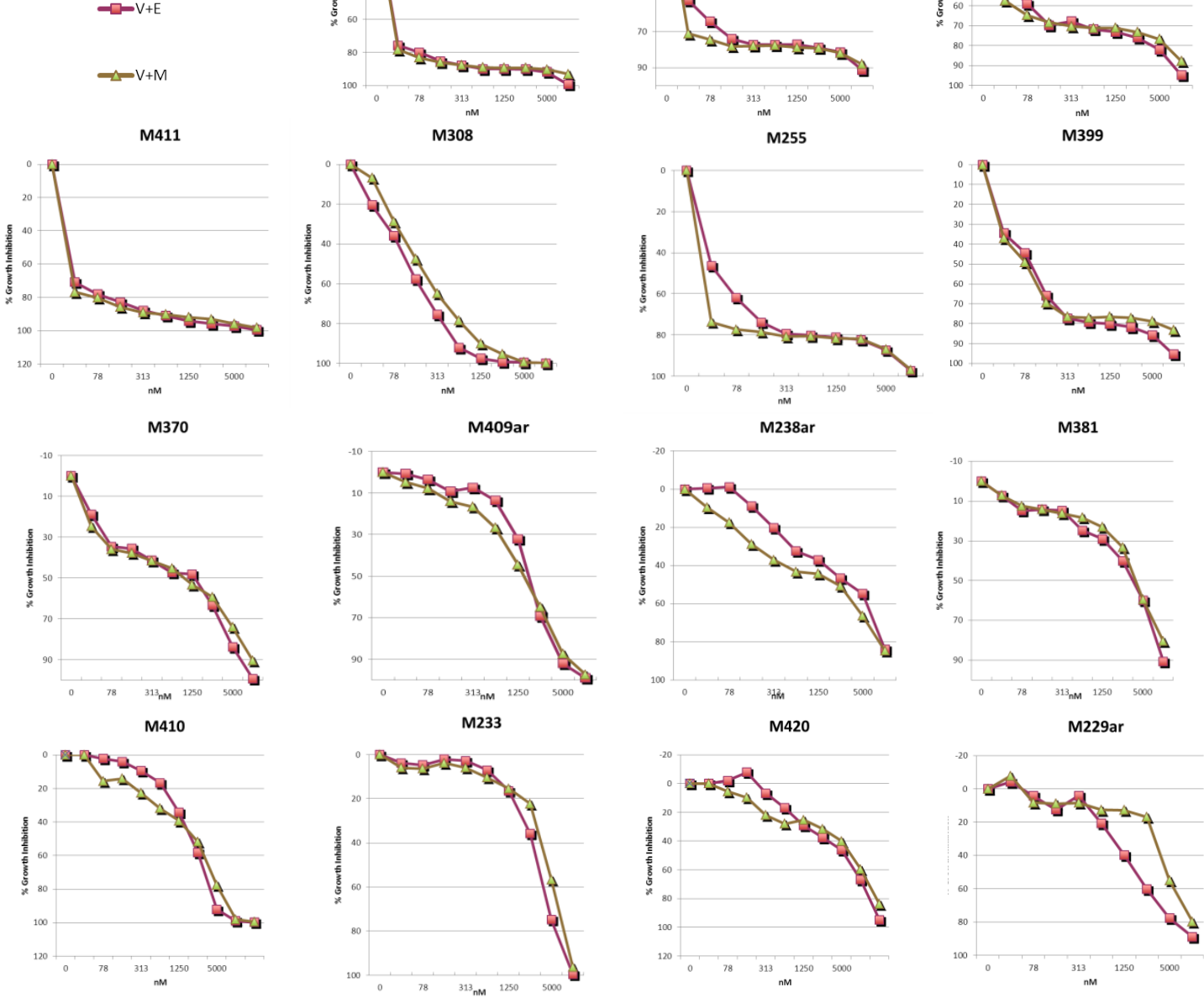

Supplement: Supplementary file 5 — Additional file 5: Figure S5: Combinatorial effect of Vemurafenib with SCH772984 or Trametinib. Percent growth inhibition of BRAF mutant cell lines. After 120 hours treatment with 0–10 μM vemurafenib (squares) combined with 0-10 uM SCH722984 (circles), or 0–10 μM vemurafenib combined with 0-1 μM trametinib, cell viability was determined by bioluminescence assay. Results are representative data in duplicate from two independent experiments (n = 6). (PDF 348 KB) [file 12943_2014_1396_MOESM5_ESM.pdf]
