# Supplementary material for: Antitumor activity of the ERK inhibitor SCH722984 against BRAF mutant, NRAS mutant and wild-type melanoma
Source: Mol Cancer. 2014 Aug 20;13:194. doi: 10.1186/1476-4598-13-194 (PMC4155088; doi:10.1186/1476-4598-13-194)

Supplemental Figure 3

A

Average of IC 50 (nM)				
Cell lines		ERKi +AKTi	ERKi + mTORi	ERKi
BRAF	M229	20.8	5.5	29.4
	M297	0.1	0.1	3.0
	M370	517.0	2055.2	2639.8
	M397	0.8	0.1	9.1
	M411	24.5	16.0	112.7
	M249	192.6	69.9	273.6
	M409	88.5	77.6	111.6
	M409AR1	854.5	1375.7	2540.5
	M233	781.3	255.6	2746.1
	M308	486.5	392.0	1080.9
	M255	162.1	81.4	264.5
NRAS	M207	9.9	3.4	37.4
	M202	135.2	24.4	230.9
	M412-A	219.7	199.2	432.7
	M244	647.4	303.4	1190.2
	M245	286.0	209.5	445.5
	M311	18.7	105.4	234.1
	M408	66.5	0.1	120.3
	M412-B	115.1	26.2	176.2
	M296	205.8	49.7	530.5
	WM1366	168.4	44.3	342.0
	SKMEL-173	36.7	19.9	80.0
WT	M230	245.4	97.9	529.4
	M418	197.0	70.7	264.3
	PB	90.9	117.4	294.4

B

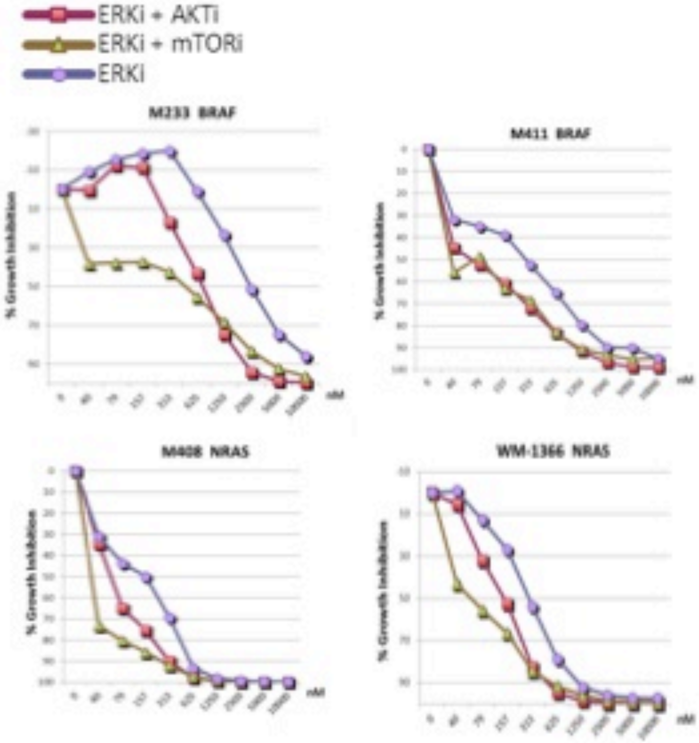

C

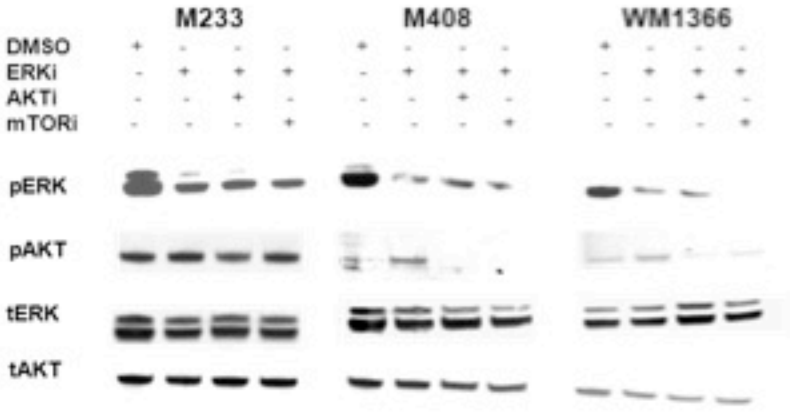

Supplement: Supplementary file 3 — Additional file 3: Figure S3: Effects of SCH722984 combined with MK-2206 (AKT inhibitor) and MK-8669 (mTOR inhibitor) in BRAF mutant cell lines. A. IC50s of SCH722984 alone or in combination with MK-2206 or MK-8669. After 120 hours treatment with 0–10 μM SCH722984, SCH722984+ MK-2206 or SCH722984+ MK-8669, cell viability was determined by bioluminescence assay. Results are representative data in duplicate from three independent experiments (n = 6). B. Percent growth inhibition for two BRAF-mutant melanoma cell lines (M233 and M411) and two NRAS-mutant cell lines (M409 and WM1366). After 120 hours treatment with 0–10 μM SCH772984 + MK2206 (ERKi + AKTi, squares), SCH722984 + MK-8669 (ERKi + mTORi, triangles), or the SCH772984 (ERKi, circles), cell viability was determined by bioluminescence assay. Results are representative data in duplicate from three independent experiments (n = 6). C. Effect ERK- inhibition alone or the combination with AKT/mTOR inhibitors on MAPK signaling. Cell lines were treated with DMSO (control, C), 1 uM SCH722984 (ERKi, E) or the combination of SCH722984+ MK-2206 and SCH722984+ MK-8669 at 1 uM for 24 hours. Western blots analyzed for phospho- and total ERK1/2, AKT and actin as loading control. (PDF 78 KB) [file 12943_2014_1396_MOESM3_ESM.pdf]
