# Supplementary material for: Antitumor activity of the ERK inhibitor SCH722984 against BRAF mutant, NRAS mutant and wild-type melanoma
Source: Mol Cancer. 2014 Aug 20;13:194. doi: 10.1186/1476-4598-13-194 (PMC4155088; doi:10.1186/1476-4598-13-194)

Supplemental Figure 6

A

Average of IC 50 (nM)					
Cell lines		ERKi +AKTi	ERKi + mTORi	ERKi	
BRAF	M229	20.8	5.5	29.4	
	M297	0.1	0.1	3.0	
	M370	517.0	2055.2	2639.8	
	M397	0.8	0.1	9.1	
	M411	24.5	16.0	112.7	
	M249	192.6	69.9	273.6	
	M409	88.5	77.6	111.6	
	M409AR1	854.5	1375.7	2540.5	
	M233	781.3	255.6	2746.1	
	M308	486.5	392.0	1080.9	
	M255	162.1	81.4	264.5	
NRAS	M207	9.9	3.4	37.4	
	M202	135.2	24.4	230.9	
	M412-A	219.7	199.2	432.7	
	M244	647.4	303.4	1190.2	
	M245	286.0	209.5	445.5	
	M311	18.7	105.4	234.1	
	M408	66.5	0.1	120.3	
	M412-B	115.1	26.2	176.2	
	M296	205.8	49.7	530.5	
	WM1366	168.4	44.3	342.0	
	SKMEL-173	36.7	19.9	80.0	
WT	M230	245.4	97.9	529.4	
	M418	197.0	70.7	264.3	
	PB	90.9	117.4	294.4	

B

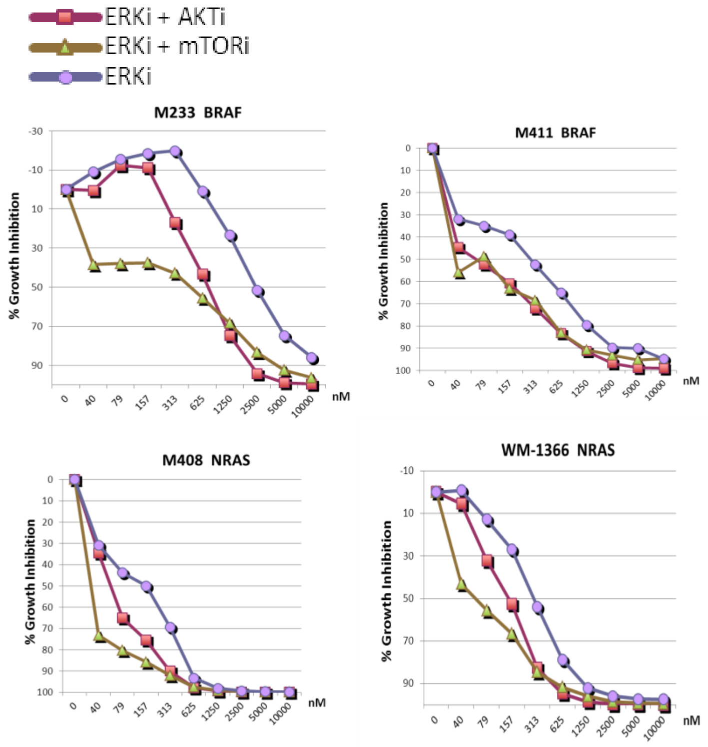

C

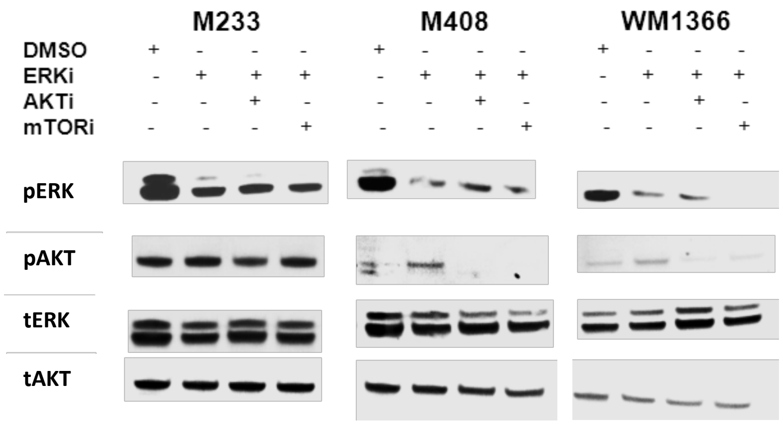

Supplement: Supplementary file 6 — Additional file 6: Figure S6: Effect of SCH722984, vemurafenib or the combination on cell cycle progression and apoptosis in BRAF-mutant melanoma cell lines. A sensitive cell line (M263), intermediate sensitivity (M255) and resistant to SCH722984 (M370) were exposed to DMSO as vehicle control (ControL), 1 μM vemurafenib (Vemurafenib), SCH722984 (ERKi), 50nM trametinib (Trametinib), the combination of 1 μM vemurafenib + 1 μM SCH722984 (V + E) or the combination of 1μMvemurafenib + 50nM trametinib (V + T) for 48 hours. A. Cell cycle progression example for M255 was tested by DAPI staining solution and induced apoptosis by cleaved PARP (PARP-Ax700). B. Apoptosis in response to MAPK inhibitors. Percentage of apoptotic cells positive for cleaved PARP (PARP-Ax700) in this three melanoma cell lines. B. Quantitative analysis of the cell cycle progression by DAPI staining using flow cytometry shows the percentage of cells in sub-G0, G0/G1, S phase, or G2/M. (PDF 199 KB) [file 12943_2014_1396_MOESM6_ESM.pdf]
